# Medical Practice and the Companies Acts

**Published:** 1905-06-03

**Authors:** 


### The Hospital.

A Journal of

The Medical Sciences and Hospital Administration.

Vol. XXXVIII.—No. 975. SATURDAY, JUNE 3, 1905.

### Medical Practice and the Companies Acts.

If we were asked to point out a reform which, perhaps more than any other, would be conducive to good government in the United Kingdom, it would be the establishment of some machinery by which existing law should be periodically inspected and overhauled. A standing departmental committee for this purpose might reasonably be attached to each of the chief Government offices, and the head of each department might reasonably be asked to introduce, whenever necessary, a short amending Bill for the correction of obvious abuses as to which no question of principle was involved. Such Bills might be referred, as a matter of course, to the Standing Committee on Law; and, as no party question would be involved in them, might be accepted by Parliament on that Committee's recommendation, and passed through the successive stages with the smallest possible delay. There are at present scores of reforms in matters of detail as to the necessity for which no two opinions can be entertained, but which, under the operation of our present Parliamentary system, are indefinitely postponed in order to afford time for idle chatter about so-called party and political differences to which no person, except the actual players of the game, can attach the smallest importance. The government of the country is carried on, not for the benefit of the lieges, but as a mere contest between the "ins" and the "outs."

Few subjects can afford a better illustration of this position than the present state of the law relating to limited joint-stock companies, concerning which Dr. McAlister, the new president of the General Medical Council, presented an elaborate report to the Council at its meeting on the 23rd instant. It may be regarded as the settled law of the country that it is a punishable offence for any individual, not possessing any registrable medical qualification, to use any title signifying that he does possess one; and it is common knowledge that, under this law, a certain number of offenders have been subjected to penalties of a deterrent character. Some of these persons discovered that it would not be unlawful for a company registered under the Companies Acts to do what would be punishable in an individual; and hence many of them who had been fined as impostors converted their several quack medical or dental businesses into companies, obtaining the necessary seven shareholders from among their families or servants, and continued their operations not only without dread of the law, but under the safe shelter afforded by enactments which had been framed for the fulfilment of totally different purposes. In the words of Dr. MacAlister's report, the servants of such a company may be, and have been, by means of lectures, pamphlets, and advertisements, placed before the public in such a manner that persons needing professional assistance are led to suppose that they are capable of administering skilled medical or dental treatment. Such servants may display great ignorance or be guilty of malpractice, and even be censured by a coroner's court, but no legal remedy is available against them. They are the irresponsible agents of an irresponsible company. The company itself has committed no offence known to the law, and thus protection is afforded to unqualified persons actually greater than that enjoyed by the qualified practitioner, who is required to possess a reasonable amount of professional knowledge and to exercise reasonable professional care and skill.

The questions hence arising have now for six or seven years been constantly under the notice of Parliament and of the Executive, but nothing has been done towards their solution. In 1899 the General Medical Council was so far successful in demonstrating the existence of a crying evil, that a short Bill was introduced by the Lord Chancellor in the House of Lords, the object of which was to render companies equally liable with individuals to the provisions of the Medical and Dentists Acts. This Bill was, by Committee of the House of Lords, placed as two clauses in the Companies Acts Amendment Bill then before the House. From this Bill the two clauses were expunged in the Committee stage in the House of Commons, after the Standing Committee on Trade had decided by a considerable majority to retain them. They were dropped on the ground that they were not germane to the general tenor of the Bill, the objects of which were financial. It would surely have been difficult, after the distinct approval of the House of Lords and of the Standing Committee, to find a more foolish or a more frivolous excuse. It has, nevertheless, been substantially adopted this year, as a ground for inaction, by a Departmental Committee of the Board of Trade, sitting to consider the operation of certain sections of the Companies Acts. In the meantime, however, a gleam of light has come to us from Ireland, where the Master of Rolls, at the instance of the Attorney-General, has dissolved a fraudulent company formed to carry on the business of dentistry, and has given costs against the defendants. In this case the promoter of the company was named Appleton, and had no dental qualification, but he styled his company "Mr. Appleton, Surgeon-dentist, Limited." As a consequence of this decision, the Privy Council has intimated to the General Medical Council that, in any case calling for it, the Attorney-General for England would probably take similar action at the instance of a proper relator; and it may therefore be regarded as probable that, after more or fewer costly trials, the law itself will be brought into harmony with the requirements of civilisation.

